# Supplementary material for: Multimorbidity and complex multimorbidity in Brazilian rural workers
Source: PLoS One. 2019 Nov 19;14(11):e0225416. doi: 10.1371/journal.pone.0225416 (PMC6863555; doi:10.1371/journal.pone.0225416)
Supplement: S1 Table — (DOCX) [file pone.0225416.s002.docx]

**DATABASE VARIABLE CODE**

| **Variable** | **Code in the database** |
| --- | --- |
| **Sex** |  |
| Male | 0 |
| Female | 1 |
| **Age Group** |  |
| Up to 29 years | 0 |
| 30 to 39 years | 1 |
| 40 or more | 2 |
| **Race / Color** |  |
| White | 0 |
| Non-White | 1 |
| **Marital status** |  |
| Not married | 0 |
| Married/Living with partner | 1 |
| Separated/Divorced/Widowed | 2 |
| **Schooling** |  |
| Less than 4 years | 0 |
| 4 to 8 years | 1 |
| More than 8 years | 2 |
| **Socioeconomic class** |  |
| Class A or B | 0 |
| Class C | 1 |
| Class D or E | 2 |
| **Land ownership** |  |
| Owner | 0 |
| Non-Owner | 1 |
| **Type of occupational contact with pesticide** |  |
| Direct | 1 |
| Indirect/Non-Contact | 2 |
| **Total number of pesticides used** |  |
| None | 0 |
| 1 to 5 types of pesticides | 1 |
| More than 5 pesticides | 2 |
| **Use of PPE** |  |
| Do not use PPE/Incomplete PPE | 1 |
| Complete PPE | 2 |
| Without direct contact | 3 |
| **Frequency of contact with pesticide** |  |
| Daily/Weekly | 1 |
| Monthly/Yearly | 2 |
| Without contact | 4 |
| **Smoking** |  |
| Non-smoker | 0 |
| Smoker or ex-smoker | 1 |
| **Practices physical activity** |  |
| No | 0 |
| Yes | 1 |
| **Alcohol consumption** |  |
| Does not consume | 0 |
| Consumes | 1 |
| **Medical diagnosis of poisoning by pesticides** |  |
| Yes | 0 |
| No | 1 |
| **Waist circumference** |  |
| Without metabolic risk | 0 |
| Increased metabolic risk | 1 |
| **Health self-assessment** |  |
| Good/ Very good | 1 |
| Fair/Poor | 2 |
| **Multimorbidity** |  |
| Yes | 1 |
| No | 0 |
| **Complex Multimorbidity** |  |
| Yes | 1 |
| No | 0 |
| **Missing Values** | 9999 |
